# *Mycobacterium leprae* Slide Positivity at Felege Hiwot Comprehensive Specialized Hospital, Northwest Ethiopia: Seven Years Retrospective Study

**DOI:** 10.1002/hsr2.70737

**Published:** 2025-04-18

**Authors:** Mulat Erkihun, Ayenew Assefa, Biruk Legese, Andargachew Almaw, Ayenew Berhan, Birhanu Getie, Teklehaimanot Kiros, Alemie Fentie, Yenealem Solomon, Bekele Sharew, Zelalem Asmare, Bayu Ayele

**Affiliations:** ^1^ Department of Medical Laboratory Sciences College of Health Sciences, Debre Tabor University Debre Tabor Ethiopia; ^2^ Department of Medical Laboratory Sciences College of Medicine and Health Sciences, Injibara University Injibara Ethiopia; ^3^ Laboratory Service Unit Felege Hiwot Comprehensive Specialized Hospital Bahir Dar Ethiopia

**Keywords:** acid fast bacilli, leprosy, *Mycobacterium leprae*

## Abstract

**Background:**

*Mycobacterium leprae* is the cause of leprosy, a disease whose severity worsens over time. It affects the nerves of the extremities, the integumentary systems, the linings of appendages like the nose, and the upper respiratory tract. This study aimed to determine the of *M. leprae* slide positivity in Felege Hiwot Comprehensive Specialized Hospital, Northwest Ethiopia.

**Method:**

Patients’ record from January 2017 to December 2023 was reviewed and extracted from laboratory registration book. Socio‐demographic data like age, sex, and confirmed cases were collected carefully. Data were cleaned, coded, entered and analyzed with statistical package for social sciences version 23.

**Result:**

The prevalence of *M. leprae* was 21.8% (138/633). Among *M. leprae* skin slit acid‐fast positive‐registered cases, 91.3% (126/138) were males and 8.7% (12/138) were females. The majority of *M. leprae* slide positive cases (78.3%, 108/138) were detected from patients in the age range of 15–46 years old. Based on this study finding, male sex is significantly associated with *M. leprae* slide positivity (AOR = 6 [1.8–15.4], 95% CI, *p* = 0.001).

**Conclusion:**

The prevalence of *M. leprae* slide acid‐fast bacilli positivity is relatively high. The high prevalence of *M. leprae* slide positivity, particularly among males, underscores the importance of targeted case detection strategies in this demographic to control leprosy. This study indicated that acid‐fast bacilli positivity of *M. leprae* is higher in males than females.

## 1 Introduction

Leprosy (Hansen's disease) is a chronic, progressive bacterial disease caused by *Mycobacterium leprae*. It affects the nerves of the extremities, the skin, the coating of the nose, and the upper respiratory tract. Leprosy brings nerve damage that results in peripheral sensory loss and muscle weakness If it isn't taken care of, it can cause severe scarring, deformities, and considerable disability. Dr. Gerhard Henrik Armauer Hansen's discovery of *M. leprae* established that leprosy is caused by a bacterium, dispelling the myths that it was inherited, a nuisance, or a sin [1, 2].

*M. leprae* is among nine Mycobacteria that have an effect on skin [3]. *M. leprae* cannot be transmitted via handshakes or touch because the bacterium cannot cross the skin. Instead, it is considered to be spread via infected droplets. There are also contradicting ideas about contact‐mediated transmission [1, 4].

The known reservoir for leprosy is man. Scholars advocate that *M. leprae* is spread from person to person by nasal secretions or droplets. However, it is not extremely infectious like flu and other infections that are droplet‐mediated. Daily contact with an infected person (including handshakes and hugs) does not result in disease transmission. The main route of transmission is by droplets of nasal mucus while sneezing, but this happens only at the early stage of the disease, even though it is not contagious [2, 5].

*M. leprae* therefore predominantly affects the skin and peripheral nerves, resulting in neuropathy and its related long‐term costs, including deformities and disabilities [6].

Based on the clinical manifestation (the number of skin lesions and presence of nerve involvement) and microscopic laboratory finding (slit‐skin smear identification of bacilli), leprosy is classified as paucibacillary (PB) or multibacillary (MB) [7, 8]. The standard treatment for leprosy includes the utilization of several (two or three) drugs, which is called multidrug therapy (MDT) [9, 10, 11]. Leprosy, like other germ‐borne diseases, can be prevented by vaccination or prophylactic antibiotics for those who have been exposed [12, 13].

*M. leprae* is an extremely variable disease, affecting all components of the population in different ways based on their immune status. The large percentage of diagnosis in children indicates elevated community transmission and the continual existence of undiagnosed cases. Children are considered the most susceptible groups for leprosy, and early‐age leprosy shows new transmission in the community as well as show how the continuing disease control program is efficient or not [14, 15]

Leprosy is a public health issue that has a direct impact on all aspects of causality due to pathogenesis and incorrect community perceptions of its causation. It is known that the common misunderstandings that the public embraces about leprosy include that it is a heritable health problem that has no treatment and is a curse from the supreme deity [16].

If leprosy patients' condition becomes complicated at diagnosis, during treatment, or after treatment, they may be admitted to improve their clinical condition [15, 17]. Especially for MB leprosy, the treatment outcome has to be monitored by a skin slit AFB investigation.

Even though the riddance or elimination of leprosy as a public health crisis was achieved worldwide in 2000 (achieving a point prevalence of under 1 per 10,000 population) and at the nation level in most countries by 2005, leprosy cases continue to occur. Over 208,619 new leprosy cases were globally reported in 2018. However, a decrease in detection rate was observed in the 2020 global report [18].

Globally, 174,087 new cases were reported in 2022; this amounts to a detection rate of 21.8 cases per million population. Compared to the 2021 total of 140,594, this represented a 23.8% increase. Every one of the six WHO regions reported a new incidence. Globally, the number of new cases fell by 19.3% between 2013 and 2022, and then by 6% until 2019. Between 2019 and 2021, the coronavirus disease 2019 (COVID‐19) pandemic reduced the number of new cases detected by about one‐third [18, 19].

Ethiopia is still reporting cases of leprosy annually, with certain regions being more affected due to factors such as poverty, limited access to healthcare, and social stigma. Despite the availability of effective MDT, leprosy is still a significant cause of disability, and delayed diagnosis often leads to irreversible nerve damage and deformities. The World Health Organization (WHO) has declared Ethiopia one of the 22 priority countries for leprosy control, highlighting the importance of sustained efforts in surveillance, early detection, and treatment (WHO, 2020). The lack of public awareness and knowledge about leprosy further complicates efforts to reduce transmission and prevent disability [20, 21].

The social implications of leprosy in Ethiopia are profound, as the disease is highly stigmatized. People affected by leprosy often face discrimination, social isolation, and reduced opportunities for employment and marriage. This stigma is rooted in both historical misconceptions about the disease and cultural beliefs, which associate leprosy with divine punishment or moral wrongdoing. As a result, individuals with leprosy may delay seeking medical care, which increases the risk of complications, transmission, and social ostracization [22, 23].

From a health systems perspective, leprosy control in Ethiopia faces significant challenges. While MDT is provided free of charge, access to healthcare services in remote and rural areas remains limited, leading to delays in diagnosis and treatment. Furthermore, there are concerns about the adequacy of health worker training, particularly in recognizing the early signs of the disease and managing complications [20, 21, 22, 23].

In Ethiopia, even if the prevalence in the country drops to a lower level and meets the WHO goal of 1 case per 10,000 population, the occurrence of new cases remains a challenge. In addition to community misconception about the disease, the disease is not acute, which does not push victims to visit health institutions; and the passive surveillance may trigger the presence of missed cases [24].

Leprosy‐related studies have been conducted in Ethiopia since the disease's introduction, but the data are only limited to specific regions of the country, and there are no data in the current study area. Therefore, this study aimed to determine the trend of *M. leprae* slide positivity at Felege Hiwot Comprehensive Specialized Hospital, Northwest Ethiopia.

## 2 Materials and Methods

### 2.1 Study Area

This retrospective study was at FHCSH in Bahir Dar, Ethiopia (January 1, 2017 to December 30, 2023). This hospital is among the largest specialized hospitals in Ethiopia, serving a population of more than 7 million people. FHCSH has 12 wards with 500 beds and approximately 1420 healthcare staff. It is 1784 m above sea level and has coordinates of latitude 11.6, longitude 37.3833 11.5742° N, and longitude 37.3614° E.

### 2.2 Study Population

The population for the study was patients examined for a *M. leprae* AFB test at Felege Hiwot Comprehensive Specialized Hospital from January 2017 to December 30, 2023.

### 2.3 Inclusion and Exclusion Criteria

The laboratory registration log records that had full information like age, sex, *M. leprae* result and year included in the study and the laboratory registration log records that didn't have full information like age, sex, *M. leprae* result and year were excluded from the study.

### 2.4 Study Design

A retrospective cross‐sectional record review was used to gather the data. Microbiologically confirmed cases of medical records from laboratory registration book (January 2017 to December 30, 2023), at the Felege Hiwot Comprehensive Specialized Hospital laboratory that were presented for skin slit microscopic examination were reviewed.

### 2.5 Data Collection and Laboratory Method

A total of 633 microbiologically confirmed cases as AFB positive or AFB negative of skin slit microscopic records of leprosy‐suspected patients in the registry “between” January 2017 and December 2023 were included in the study. The data was entered into statistical package for social sciences (SPSS) version 23 software.

From skin with nodules or from an earlobe that was an alcohol‐cleaned incision of 3–5 mm long and 2–3 mm deep by a single‐edged blade, smears of two to six were done. Detecting *M. leprae* was employed by using staining (AFB), which can be performed by either Ziehl‐Neelsen staining, which contains a primary stain (concentrated carbol fuchsinor basic fuchsin dissolved in phenol), a decolorizer, and a counter stain (Loeffler's methylene blue), or florescent microscopic technique, which contains the primary stain auramine O, decolorizer, and the quenching potassium permanganate as a counterstain or to highlight the stained organisms for easier recognition. Due to the fact that heat is utilized to penetrate the waxy cell walls of this challenging‐to‐stain organism, the procedure is likewise known as the “hot staining method.” Since the regular Ziehl‐Neelsen procedure can't readily decolorize *M. leprae's* mycolic acid coating, modified Ziehl‐Neelsen staining is the best option for demonstrating the organism. The smears were placed in a side‐up serial sequence on the staining bridge and flooded with 1% Carbol Fuchsin that had been filtered. After being steam‐heated and left to stain for 5 min, the smears were washed with water and emptied. After 5 min of decolonization with 1% acid alcohol, they were washed with water and emptied. After 1 min of counterstaining with a 0.1% methylene blue solution, they were washed with water. After letting the smear air dry, it was viewed under a microscope using an oil immersion (× 100) objective.

### 2.6 Data Analysis

The statistical package for social sciences (SPSS) version 23 software was used to analyze and present the data. The descriptive statistics and logistic regression models were employed. The association between slide bacilli positivity and socio‐demographic variables was done at 95% confidence interval (CI) and *p*‐values less than 0.05 were considered statistically significant.

## 3 Result

### 3.1 The Characteristics of the Study Cases

In total, 633 patients were sent to the TB leprosy laboratory for slide leprosy status confirmation in the last 5 years (2017–2023).

The participants were presented from all over the Amhara region. Among the patients sent to the laboratory for *M. leprae* slide status confirmation, 69.7% (441/633) were males and 30.3% (192/633) were females. The majority of patients, 70.6% (447/633) were in the age category of 15–47 years old. The minimum and maximum ages were 11 and 90 years old, respectively, with a range of 79 years. The mean age was 37 years old. Out of these patients, 96.2% (624/633) were new cases, and 3.8% (24/633) were follow‐up cases of leprosy (Table 1).

**Table 1:** The characteristics of the study cases from January 2017 to 2023.

Factors	Alternatives	Frequency (*N*)	Percentage
Sex	Male	441	69.7
	Female	192	30.3
Age category	0–14	21	3.3
	15–47	447	70.6
	48–63	108	17.1
	≥ 64	57	9.0
Patient status	New	609	96.2
	Follow‐up	24	3.8
*Mycobacterium leprae*	AFB‐positive	138	21.8
	AFB‐negative	495	78.2
Total		633	100

### 3.2 The Prevalence of *M. leprae* Among Smear Examined Patients

The prevalence of *M. leprae* from this analysis was 21.8% (138/633). Among *M. leprae* smear‐positive registered cases, 91.3% (126/138) were males and 8.7% (12/138) were females. In this study, 93.5% (129/633) of *M. leprae* smear positivity was observed in new cases sent for laboratory confirmation, while 6.5% (9/138) were follow‐up cases sent for treatment response confirmation. Among registered *M. leprae* slide‐positive cases, the highest prevalence of 78.3% (108/138) was observed in the age categories of 15–46 years old (Table 2).

**Table 2:** The prevalence of *M. leprae* among smear examined patients from January 2017 to January 2023 total suspected cases.

Factors	Alternatives	*M. leprae*		AOR (95% CI)	*p* value
		AFB‐positive (%) *N* = 138	AFB‐negative (%) *N* = 495		
Sex	Male	126	315	6 (1.8 ≥ 15.4)	0.001
	Female	12	180		
Patient status	New	129	480	2 (0.4 ≥ 10.1)	0.4
	Follow‐up	9	15		
Age category	0 ≥ 14	1	21	1	
	15 ≥ 45	89	330	5 (0.01 ≥ 22.5)	0.6
	45 ≥ 64	40	94	8 (0.01 ≥ 22.5)	0.4
	≥ 65	8	50	4 (0.01 ≥ 22.50)	0.02

**Figure 1:**
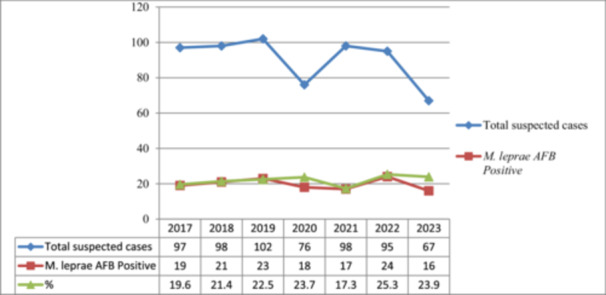
The case detection rate in consecutive years from the suspected cases (2017–2023).

According to the statistical analysis of different independent variables with *M. leprae* sex is significantly associated [AOR = 6(1.8–15.4), 95% CI, *p* = 0.001] with *M. leprae*, acid fast positivity. Male patients are six times more likely to be AFB positive for *M. leprae* than females. Moreover, the elder patients (in the age category of above 65 years) are five times likely to be AFB positive when compared with children However, variables such as patient status are not significantly related (Table 2)**.** The trained of 7 years case detection in each ear is almost similar (Figure 1).

## 4 Discussion

Leprosy is recognized as one of the neglected tropical diseases (NTDs) in Ethiopia and is among the diseases targeted for eradication through strategic planning. There are cases where it encourages stakeholders to promote policies that hinder the eradication process.

Based on this retrospective data analysis, the overall prevalence of slide acid fast bacilli positive for *M. leprae* is 21.8%. The prevalence of the current analysis is greater than the WHO standard to be detected in a population of 10,000, which should be less than 1% [25]. But comparable with the current WHO updates at which globally, 174,087 new cases were reported in 2022. This could be because the participants who are brought to the health facility have clinical manifestations, indicating that surveillance is passive in Ethiopia.

The current study has higher prevalence from the study conducted at Boru Meda General Hospital northeastern Ethiopia whose prevalence was 14.8% [26].

But the prevalence of AFB‐positive leprosy in our study, which is 21.8%, is lower than the study conducted in India, which had a prevalence of 29.6% [26]. It is possible that India is one of the countries with a high leprosy burden. Because more than 60% of global leprosy is contributed by India [27, 28]. Another study looked at 17 years of retrospective data in Rwanda, from 1995 to 2011, 266 patients were sent to the laboratory for confirmation of their slide‐microscopy leprosy status. Of them, 77 (28.9%) were slide acid fast bacilli positive, which has a higher prevalence than our analysis [29]. There was also another study conducted in Bangladesh with a prevalence of AFB positivity of 16%, which is less prevalent than our analysis [30]. The other study on the prevalence of smear‐positive leprosy in India revealed a nearly similar proportion but a slightly greater prevalence without a significant difference (26.7%) with our study, which has a 21.8% prevalence of acid‐fast bacilli detection [31]. Although WHO launched the Global Leprosy Strategy in 2016 (2016–2020) with the slogan “Accelerating toward a leprosy‐free world” [32], generally global case surveillance of *M. leprae* looks passive.

Males were found to have a higher proportion of *M. leprae* smear positives (91.3%) than females (only 8.7%). The finding is concordant with analyses that have been conducted in Rwanda, where smear‐positive cases have a 3:1 male‐to‐female ratio, but our finding is very high for males compared with females (11:1 male‐to‐female ratio) [25]. The other analysis revealed a higher proportion in males, with a male‐to‐female ratio of 2:1, with the highest prevalence in the age groups of 15–44 years [33, 34]. It is possible to conclude that males are more affected than females, which needs further investigation.

The higher susceptibility of males to *M. leprae* infection, compared to females might be influenced by a combination of biological, hormonal, and social factors. One major contributing factor is the difference in immune system function between the sexes. Research has shown that females generally have stronger innate immune responses, which may provide them with greater protection against infectious diseases, including leprosy. Estrogen, a hormone predominant in females, has been found to enhance immune responses, while testosterone, which is more abundant in males, may suppress certain aspects of immune function, making men more vulnerable to infections like leprosy. Additionally, gender differences in behavior and exposure risks also play a role in the higher incidence of leprosy in males. In many societies, males are more likely to engage in outdoor activities, work in agricultural settings, or live in environments where they are more likely to contact the bacteria, which is transmitted through respiratory droplets. These behavioral patterns increase the risk of exposure to *M. leprae*. Men also tend to seek medical attention less frequently than women, potentially leading to delays in diagnosis and treatment, which can allow the infection to progress further and contribute to a higher disease burden in males. Furthermore, genetic and cultural factors might influence the differential rates of infection between males and females. Some studies suggest that there may be genetic predispositions linked to sex chromosomes that make men more susceptible to leprosy. Additionally, in certain cultures, there may be gender‐specific stigmas or social determinants that affect how males and females interact with healthcare systems, impacting both prevention and treatment outcomes. This complex interaction of biological, environmental, and societal factors likely contributes to the higher rates of leprosy observed in males compared to females [35, 36, 37, 38, 39].

Unlike the Rwandan study, which found that the majority of *M. leprae* slide positive cases were over the age of 45, our analysis found that the majority of *M. leprae* slide positive cases (78.3%) were under the age of 45 [29]. In the current study, being elderly patient was significantly associated with the AFB positivity than the children. This could be Leprosy has a long incubation period, which can span several years or even decades. Elderly individuals might have been infected many years ago but are only showing detectable signs of infection now due to the long latency period. Children are less likely to have reached this latency period, thus showing fewer positive tests.

### 4.1 Limitation of the Study

The current study didn't contain clinical or paucibacillary leprosy. Only positive slide prevalence was given special consideration, making it difficult to determine the overall prevalence of the disease in the study area.

## 5 Conclusion and Recommendation

The proportion of positive skin slit slide acid‐fast bacilli is relatively high, indicating that more effort is needed for case detection to have a leprosy‐free generation. The high proportion of *M. leprae* slide positivity in this population, especially in men, emphasizes the need for focused case identification techniques to control leprosy. This analysis also indicated that further investigation should be conducted for biological and cultural reasons why males are more skin‐slit‐slide AFB‐positive than females. Finally, what this analysis indicated is that there are follow‐up victims who were positive for skin slit slide acid fast bacilli, which gives a clue to the further investigations for drug resistance of *M. leprae*. Generally, awareness creation, campaigns for early screening and better access to diagnostic services for at‐risk populations will bring better out come in leprosy free generation creation journey.

## Author Contributions

**Mulat Erkihun:** conceptualization, investigation, writing – original draft, methodology, validation, writing – review and editing, formal analysis, data curation, resources, project administration, visualization, software. **Ayenew Assefa:** conceptualization, writing – review and editing, validation. **Biruk Legese:** investigation, writing – review and editing. **Andargachew Almaw:** conceptualization, writing – review and editing, visualization, supervision. **Ayenew Berhan:** writing – review and editing, visualization, validation, supervision. **Birhanu Getie:** conceptualization, writing – review and editing, visualization, validation, methodology. **Teklehaimanot Kiros:** conceptualization, writing – original draft, writing – review and editing, visualization, validation, methodology, supervision. **Alemie Fentie:** conceptualization, writing – review and editing. **Yenealem Solomon:** conceptualization, writing – review and editing, validation, visualization. **Bekele Sharew:** conceptualization, writing – review and editing, visualization, validation, methodology, supervision. **Zelalem Asmare:** conceptualization, writing – review and editing, visualization, validation, methodology. **Bayu Ayele:** writing – review and editing, conceptualization, investigation, visualization, data curation.

## Ethics Statement

Ethical clearance was obtained from the Research and Ethical Review Committee of the College of Health Sciences, Debre Tabor University, with reference number: CHS/RCC/112/14. Since the study involved a retrospective record review, the requirement for informed consent was waived by institutional review board (IRB). Additionally, permission to conduct the study was obtained from Felege Hiwot Comprehensive Specialized Hospital.

## Conflicts of Interest

The authors declare no conflicts of interest.

## Transparency Statement

The lead author Mulat Erkihun affirms that this manuscript is an honest, accurate, and transparent account of the study being reported; that no important aspects of the study have been omitted; and that any discrepancies from the study as planned (and, if relevant, registered) have been explained.

## Data Availability

A summary of patient information and associated factors with *M. leprae* are included in the study in the form of texts, tables, and figures. Conclusions were also drawn from these data. Raw data can be obtained from author/s upon rational request. Basically, the corresponding author, Mulat Erkihun, could be contacted on behalf of the authors.
